# Supplementary material for: TP53 mutations and RNA-binding protein MUSASHI-2 drive resistance to PRMT5-targeted therapy in B-cell lymphoma
Source: Nat Commun. 2022 Sep 27;13:5676. doi: 10.1038/s41467-022-33137-8 (PMC9515221; doi:10.1038/s41467-022-33137-8)
Supplement: Supplementary file 1 — Supplementary Information [file 41467_2022_33137_MOESM1_ESM.pdf]

Supplementary Information:

TP53 mutations and RNA binding protein MUSASHI2 drive resistance to PRMT5-targeted therapy in B-cell lymphoma

Tatiana Erazo[1], Chiara M. Evans[1,2], Daniel Zakheim[3], Eren L. Chu[1], Alice Yunsi Refermat[3], Zahra Asgari[4], Xuejing Yang[1], Mariana Da Silva Ferreira[1], Sanjoy Mehta[3], Marco Vincenzo Russo[3], Andrea Knezevic[5], Xi-Ping Zhang[6], Zhengming Chen[7], Myles Fennell[3], Ralph Garippa[3], Venkatraman Seshan[5], Elisa de Stanchina[8], Olena Barbash[6], Connie Lee Batlevi[4], Christina S. Leslie[9], Ari M. Melnick[10], Anas Younes[4*#], Michael G. Kharas[1*#]

[1]Molecular Pharmacology Program, Experimental Therapeutics Center and Center for Stem Cell Biology, Memorial Sloan Kettering Cancer Center, New York, N, USA.
[2]Department of Pharmacology, Weill Cornell School of Medical Sciences, New York, NY, USA.
[3]Gene Editing and Screening Core, Memorial Sloan Kettering Cancer Center, New York, NY, USA.
[4]Lymphoma Service, Memorial Sloan Kettering Cancer Center, New York, NY, USA.
[5]Department of Epidemiology and Biostatistics, Memorial Sloan Kettering Cancer Center, New York, NY, USA.
[6]Epigenetics Research Unit, GlaxoSmithKline, Collegeville, PA 19426, USA.
[7]Division of Biostatistics and Epidemiology, Weill Cornell Medicine, New York, NY 10021, USA.
[8]Antitumor Assessment Core, Memorial Sloan Kettering Cancer Center, New York, NY, USA.
[9]Computational Biology Program, Memorial Sloan Kettering Cancer Center, New York, NY, USA.
[10]Division of Hematology and Medical Oncology, Sanford I. Weill Department of Medicine, Weill Cornell Medicine, New York, NY, USA

* These authors jointly supervised this work: Anas Younes and Michael G Kharas
# Corresponding Authors: (Lead contact) Michael G Kharas: kharasm@mskcc.org,
Anas Younes: anas.younes@astrazeneca.com

Supplementary figures

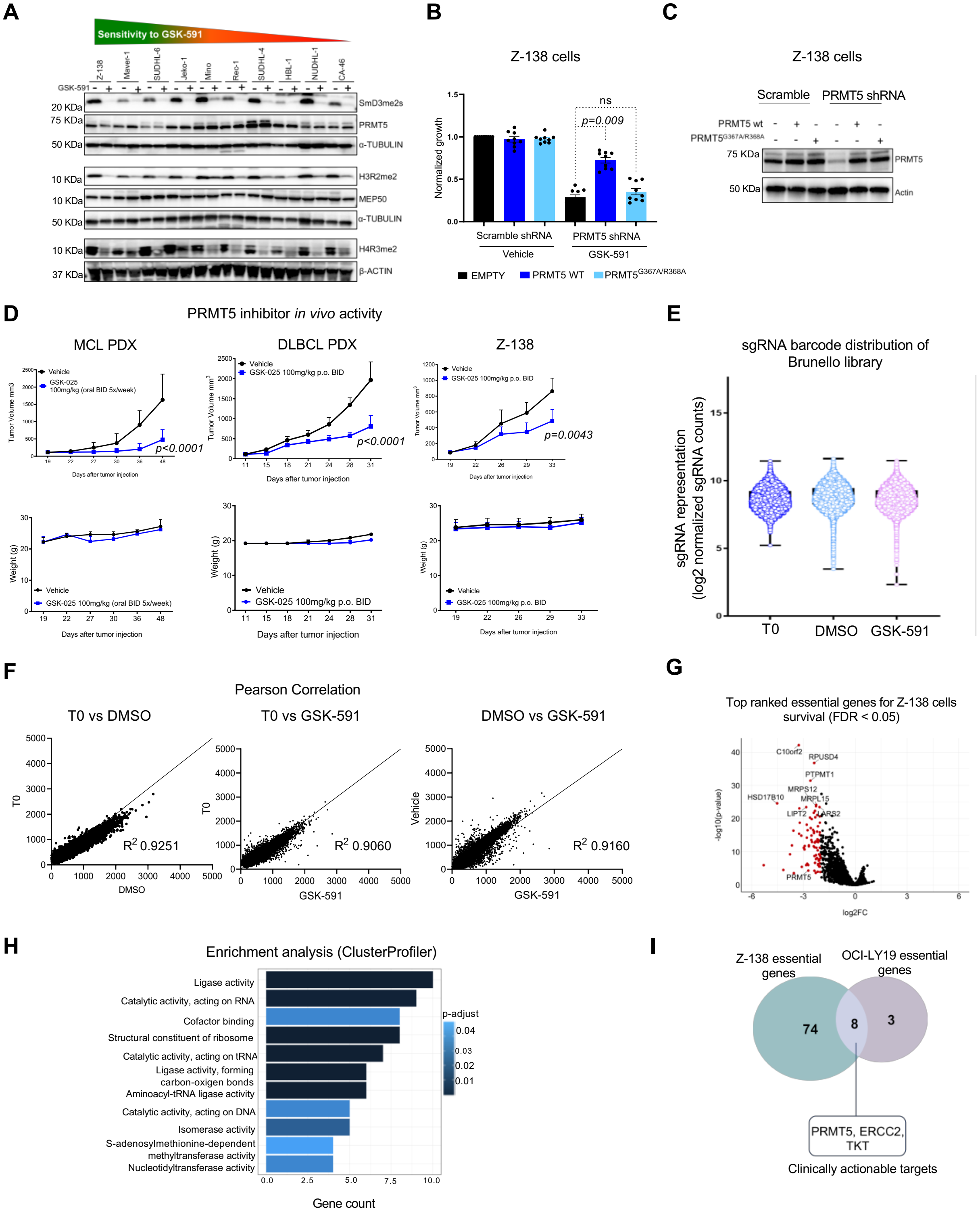

Supplementary Fig. 1. **PRMT5 is essential for lymphoma cells survival.**

A. Immunoblots showing GSK-591 inhibits PRMT5 activity in lymphoma cell lines after 48 hours of treatment. PRMT5 specific targets of PRMT5, PRMT5 and MEP50 were analyzed alpha-tubulin and beta-actin were used as loading control. **B.** Z-138 cells expressing PRMT5 shRNA or scramble shRNA were transduced with Flag-tagged PRMT5 wild type (wt) or catalytic dead double mutant ($PRMT5^{G367A/R368A}$) or Flag-empty vector. Cells were treated with GSK-591 (1 μM) and cell viability assay was performed 4 days post-transduction. Differences between groups (scramble versus PRMT5 shRNA) were calculated with the Student's test, *$p<0.05$. **C.** Immunoblot analysis of the indicated proteins from cells expressing PRMT5 shRNA or scramble. **D.** Relapsed MCL DFBL-98848 and DLBCL DFBL-75549 PDX lines and Z-138 were xenografted subcutaneously in NSG mice. After about 2 weeks, animals were randomized (n=8/group) and treated with vehicle or GSK-025, 100 mg/kg twice/day for 21 days. Data are represented as mean $\pm$ SEM. *p-values* were calculated by two-sided ANOVA. **E.** Box plot showing the sgRNA normalized read counts at Day 0 (T0) and after 8 days of treatment with GSK-591 or DMSO. Maxima = 12.4 and minimum = 2.9. **F.** Pearson correlation coefficient of the normalized sgRNA read counts from Brunello library of transduced cells at Day 0 and upon treatment with GSK-591 and DMSO. **G.** Volcano plot showing the top essential genes for Z-138 cells survival. The *x*-axis shows log2 fold change and the *y*-axis shows $-\log_{10}$ of the adjusted *P* value (adjust *p-value* <0.05). Red dots represent the genes that were significant depleted defined by adjust *p-value*<0.1 and log2FC <-2. *p-values* and $\log_2$FC were generated using the Wilcoxon-Mann-Whitney test in the CAMERA function. **H.** Pathway enrichment analysis of essential genes for Z-138 lymphoma cells survival. To identify the Gene ontology categories and corresponding *p-values* a one-sided version of Fisher's test was performed. **I.** Overlap of dropout genes in Z-138 and OCI-LY19 identified by the CRISPR screens to determine the potentially actionable targets, that are the genes that encoded for proteins with available inhibitors reported in the DepMap database.

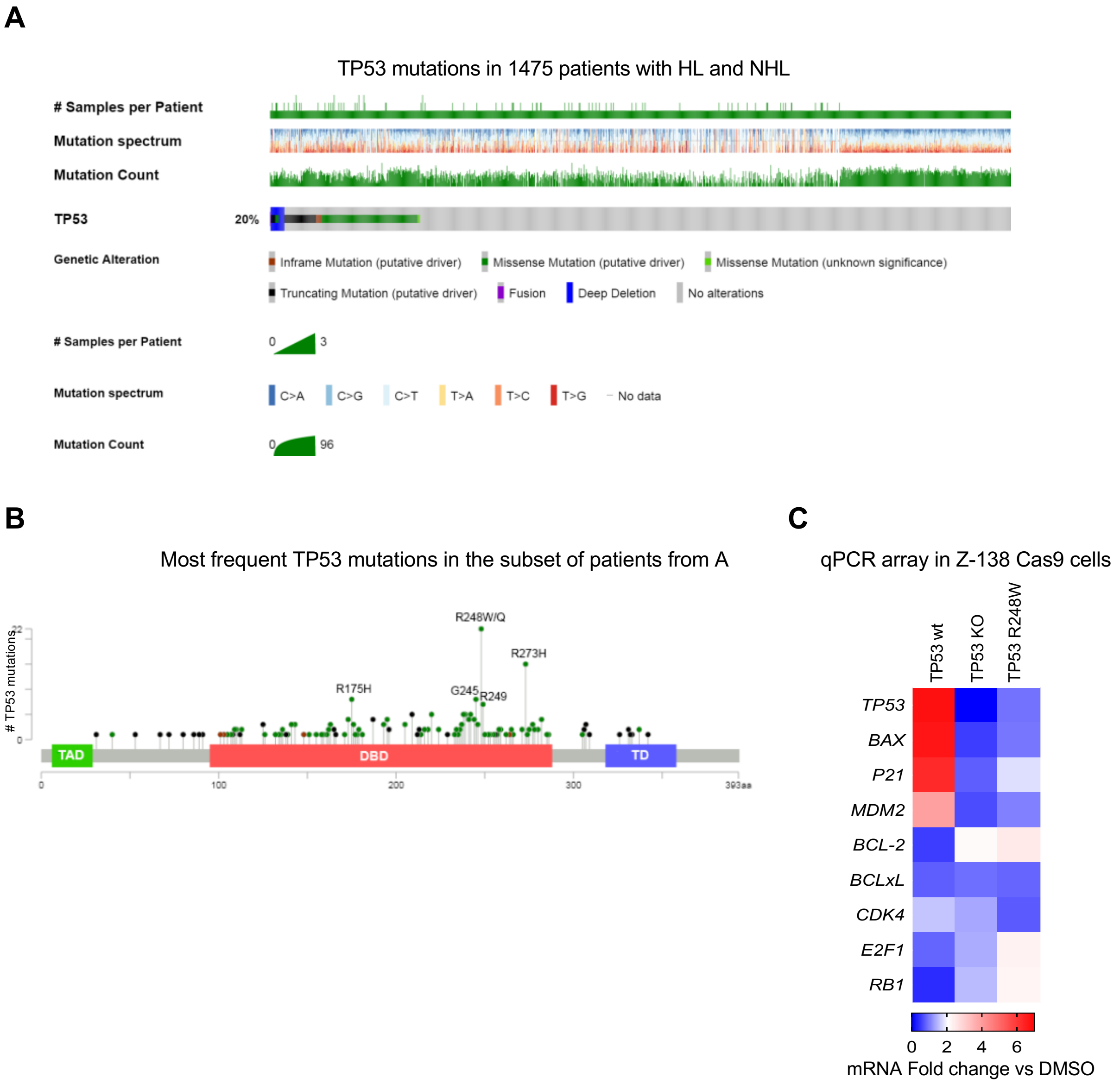

Supplementary Fig. 2. ***$TP53^{R248W/Q}$* is the most common mutation in lymphoma patients.**
A. Oncoplot showing distribution of mutations in *TP53* in 1475 patients diagnosed with HL and NHL. Data obtained from MSKCC cBioportal database after targeted next-generation sequencing using MSKCC HemePACT platform. The percentage of tumors with alterations is indicated. **B.** Lollipop plot showing that *$TP53^{R248W/Q}$* is the most frequent mutation, followed by R273H, R175H, G245 and R249 in the subset (20%) of lymphoma patients from C that carries mutations in *TP53*. TP53 domains: TAD (trans-activation domain), DBD (DNA-binding domain) and TD (tetramerization domain). **C.** Heat map showing the effect of GSK-591 (1μM) treatment for 48 h on P53 pathway, determined by a PCR-directed array in Z-138 *TP53* wt (parental) and P53 KO1 and *$TP53^{R248W}$* (Clone 2). Fold change values are depicted in a colorimetric scale from blue (low) to red (high) with respect to control (DMSO). The experiments were carried out in triplicate.

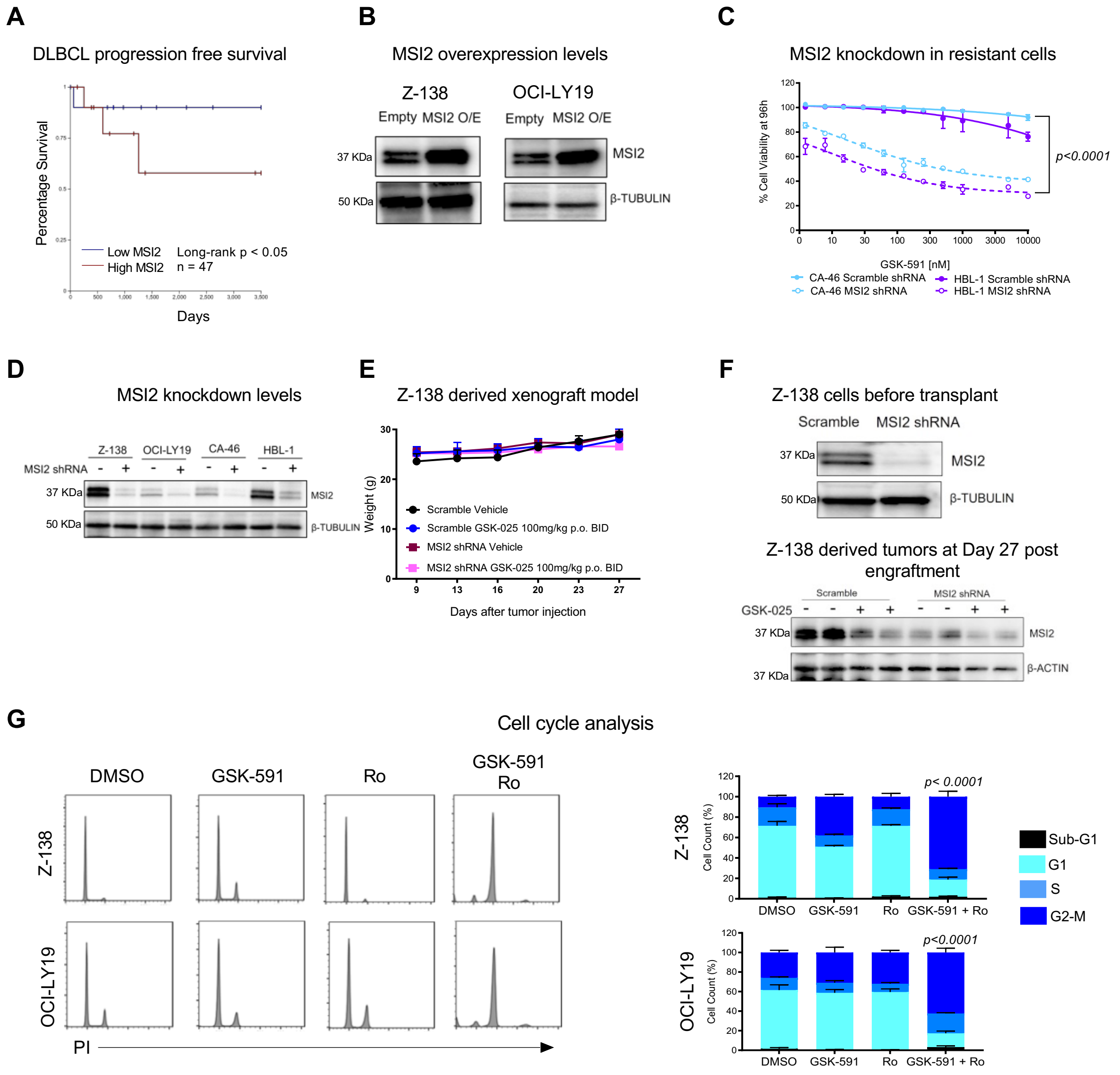

Supplementary Fig. 3. MSI2 knockdown sensitizes cells to PRMT5 inhibitor and combination of GSK-591 and Ro induced cell cycle arrest.

A. Progression-free survival of DLBCL patients with different levels of expression of MSI2 (TCGA). **B.** Immunoblot analysis of MSI2 in Z-138 and OCI-LY19 cells overexpressing empty vector or FLAG-MSI2 96h post-transduction. **C.** HBL-1 and CA-46 cells were transduced with lentivirus expressing control shRNA(shCtrl) or shRNA targeting MSI2. Error bars represent SD of three different experiments. *p<0.01. **D.** Immunoblot analysis of MSI2 knockdown in Z-138, OCI-LY19, HBL-1 and CA-46 cells transduced with shRNA(shCtrl) or shRNA targeting MSI2. **E.** Variations of body weight over time are shown from mice treated with vehicle or GSK-025, 100 mg/kg twice/day for 21 days. **F.** Immunoblot analysis of MSI2 knockdown in Z-138 transduced with scramble or MSI2 shRNA previous transplant into NSG mice and tumor lysates from two mice per condition were analyzed at Day 27 post-engraftment. **G.** Drug combination induced G2/M cell-cycle arrest. Effect of the 5uM GSK-591 and Ro alone or in combination for 24h on cell-cycle fractions in lymphoma cell lines, Z-138 and OCI-LY19. Error bars represent S.E.M. of triplicate experiments.

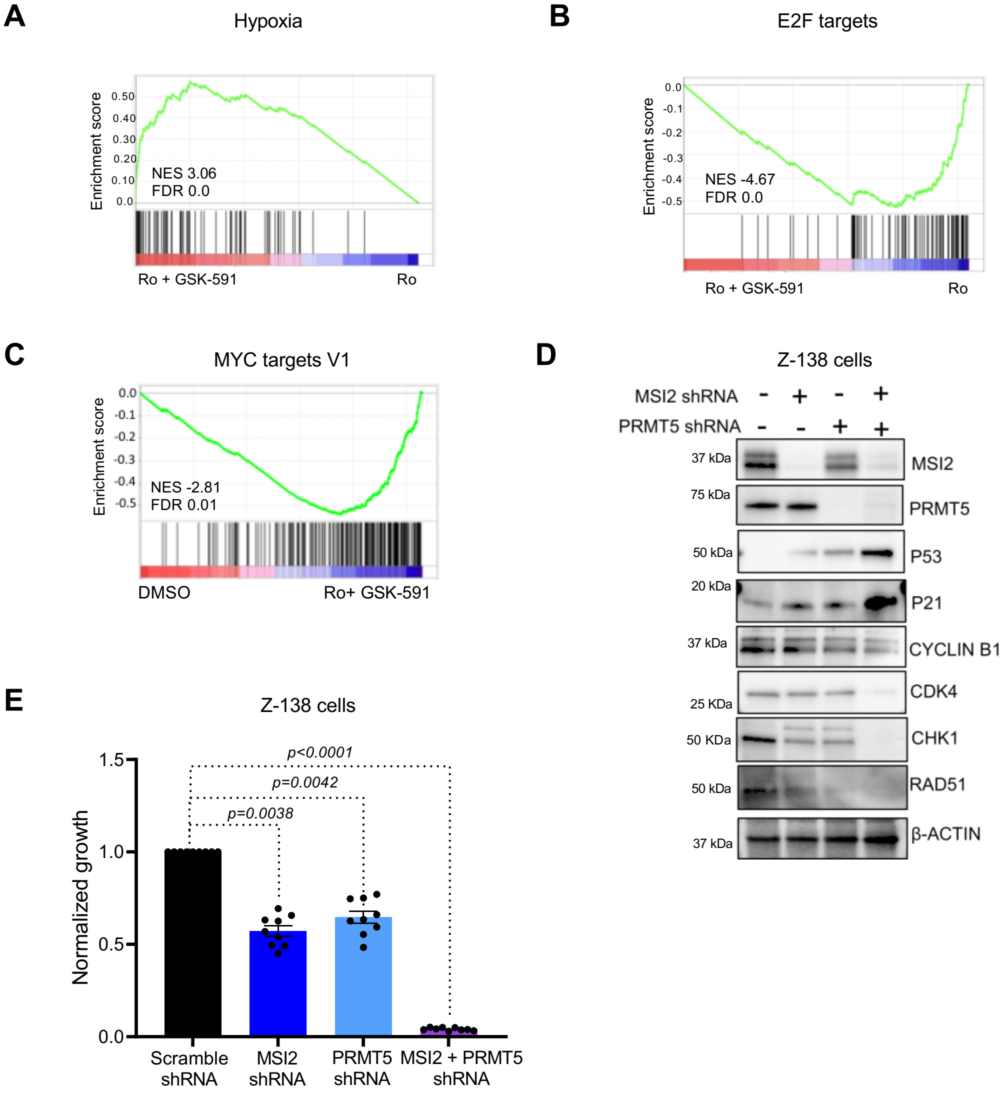

Supplementary Fig. 4. Dual targeting of MSI2 and PRMT5 induced transcriptional changes in multiple pathways. A. GSEA enrichment plots for the pathway gene set 'Hypoxia' of mRNA expression changes observed upon the combination of Ro and GSK-591 vs. Ro in Z-138 cells. **B.** GSEA enrichment plots for the pathway gene set 'E2F targets' of mRNA expression changes observed upon the combination of Ro and GSK-591 vs. Ro in Z-138 cells. **C.** GSEA enrichment plots for the pathway gene set 'MYC TARGETS V1' of mRNA expression changes observed upon the combination of Ro and GSK-591 vs. control in Z-138 cells. **D.** Immunoblot analysis of the indicated proteins from Z-138 cells transduced with shRNAs targeting PRMT5 and MSI2. **E.** MSI2 and PRMT5 genetic depletion reduced cell growth. Z-138 cells were transduced with lentivirus expressing shRNA targeting PRMT5, MSI2 or scramble. Cell viability was assessed 5 days after transduction and puromycin selection. Experiments were performed in triplicates and *p-values* were determined using two-tailed *t* test.

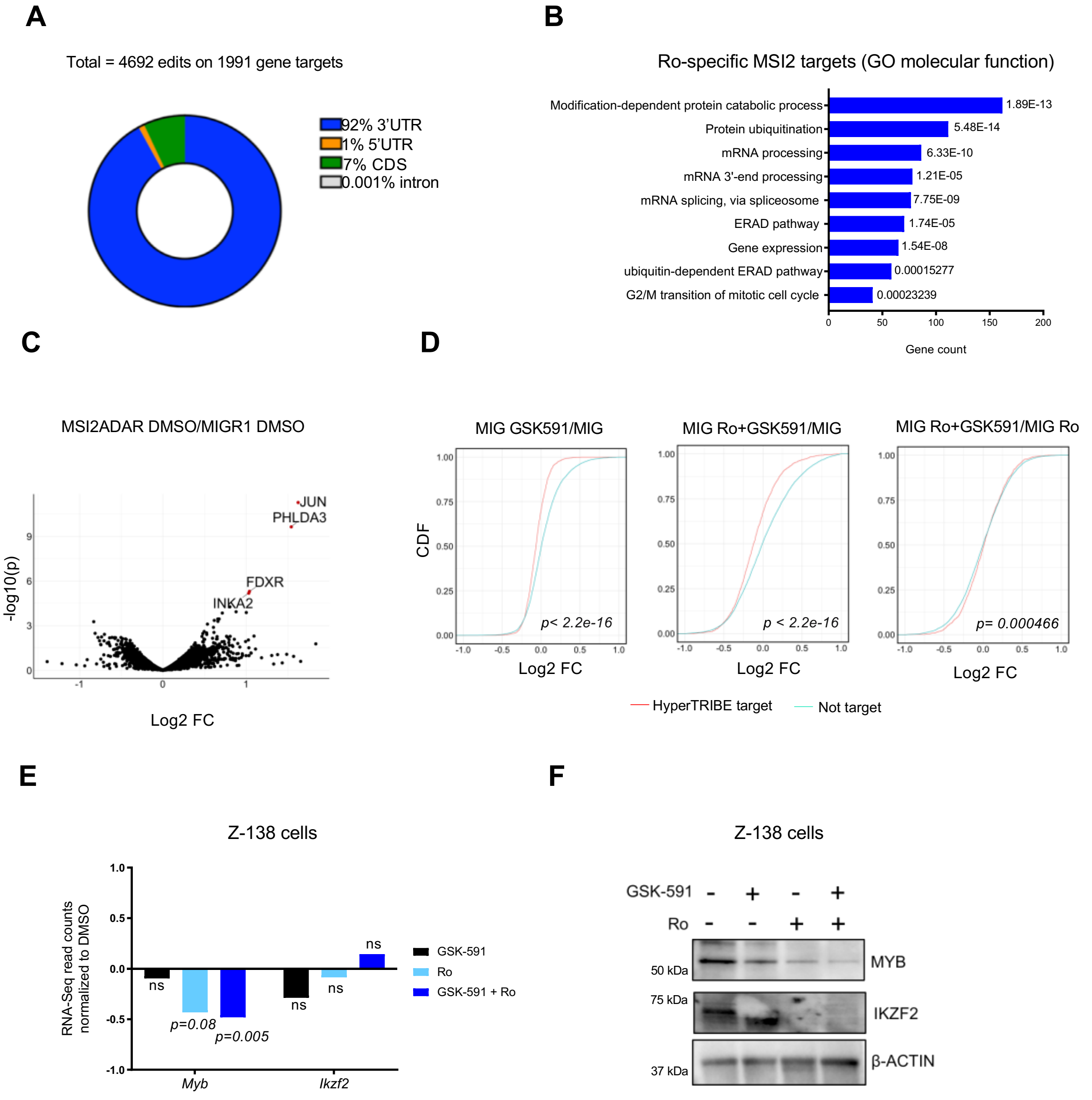

Supplementary Fig. 5. MSI2-ADAR binds to the 3'UTRs and does not induce changes in the transcriptome. A. Total number of MSI2-HyperTRIBE significant edit sites, target genes, and distribution of sites on the genes in Z-138 cells from three HyperTRIBE experiments. **B.** Top significant enriched GO molecular functions in the Ro-specific targets using ENRICHR analysis. **C.** Differential expression (DESeq2) analysis of MSI2-ADAR overexpression in Z-138 cells. Red dots represent genes with significant differential expression in MSI2-ADAR versus MIG control defined as adjusted *p-value* <0.1. **D.** CDF plots showing the distribution of mRNA abundance changes in MSI2 HyperTRIBE targets upon the indicated conditions. A two-sided KS test was used to calculate the *p-values*. **E.** mRNA expression of *MYB* and *IKZF2* from RNA-Seq experiment Fig. 4E, n = 3. Adjust *p-values* are indicated. **F.** Immunoblot analysis of MYB and IKZF2 from Z-138 cells treated with 5uM of Ro and/or GSK-591 for 24h.

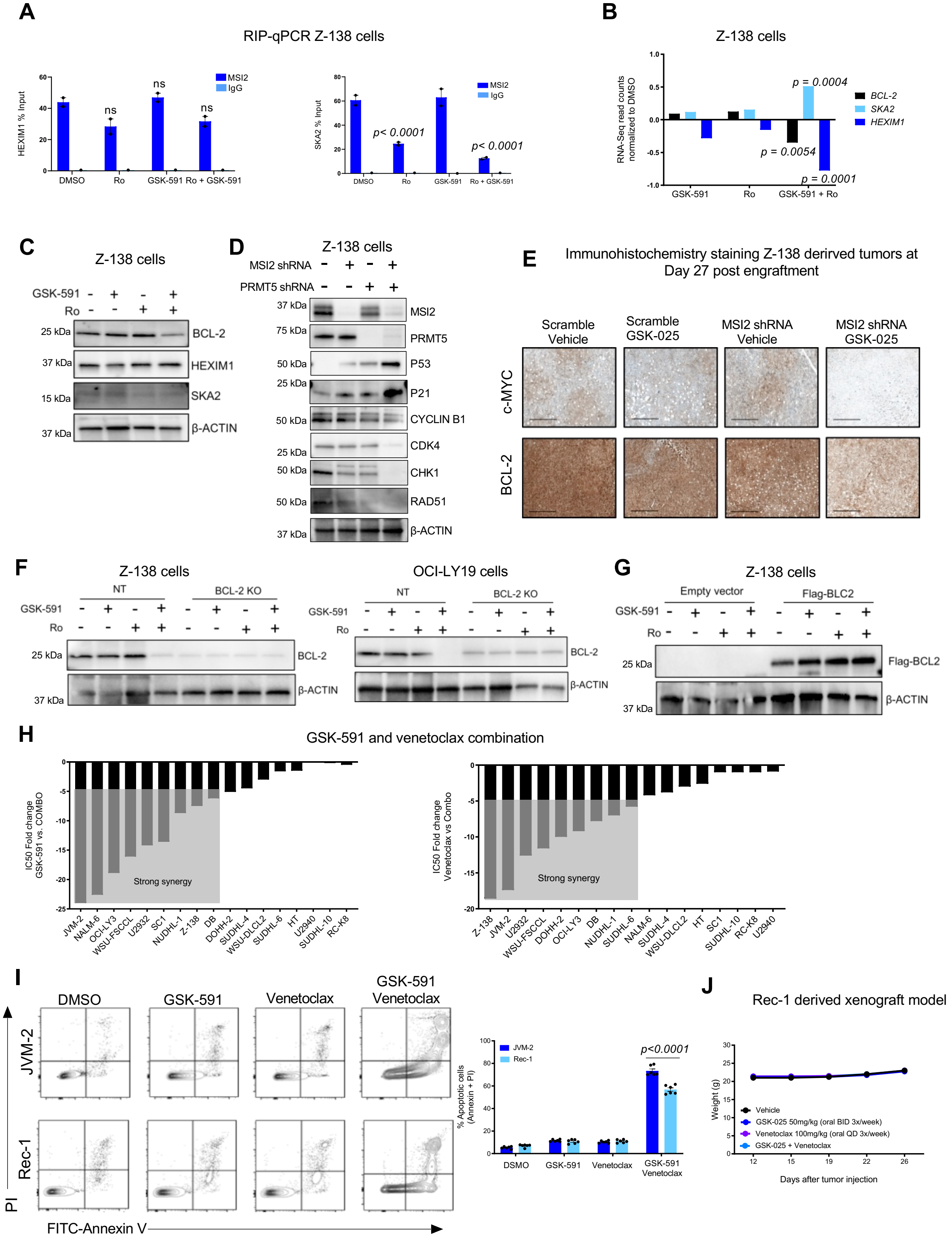

A
RIP-qPCR Z-138 cells
HEXIM1 % Input
MSI2
IgG
ns
DMSO
Ro
GSK-591
Ro + GSK-591
SKA2 % Input
p< 0.0001
B
Z-138 cells
RNA-Seq read counts normalized to DMSO
BCL-2
SKA2
HEXIM1
p = 0.0004
p = 0.0054
p = 0.0001
GSK-591 + Ro
C
Z-138 cells
BCL-2
HEXIM1
SKA2
β-ACTIN
D
MSI2 shRNA
PRMT5 shRNA
MSI2
PRMT5
P53
P21
CYCLIN B1
CDK4
CHK1
RAD51
E
Immunohistochemistry staining Z-138 derirved tumors at Day 27 post engraftment
Scramble Vehicle
Scramble GSK-025
MSI2 shRNA Vehicle
MSI2 shRNA GSK-025
c-MYC
F
OCI-LY19 cells
NT
BCL-2 KO
G
Empty vector
Flag-BLC2
Flag-BCL2
H
GSK-591 and venetoclax combination
IC50 Fold change GSK-591 vs. COMBO
IC50 Fold change Venetoclax vs Combo
Strong synergy
I
DMSO
Venetoclax
GSK-591 Venetoclax
JVM-2
Rec-1
PI
FITC-Annexin V
% Apoptotic cells (Annexin + PI)
p<0.0001
DSMO
J
Rec-1 derived xenograft model
Weight (g)
Vehicle
GSK-025 50mg/kg (oral BID 3x/week)
Venetoclax 100mg/kg (oral QD 3x/week)
GSK-025 + Venetoclax
Days after tumor injection

Supplementary Fig. 6. SKA2 and BCL-2 are targets of MSI2 and combination of GSK-591 and venetoclax induced apoptosis.

A. qRT-PCR of recovered RNA from *MSI2* RNA-IP from Z-138 cells treated with 5μM GSK-591 and/or Ro for 24h. *HEXIM1* and *SKA2* mRNA enrichment are shown as the percentage (IP/input) and normalized to DMSO ± SD of two independent experiments. IgG served as a non-specific binding control. **B.** BCL-2 mRNA expression from RNA-Seq experiment Figure 4E, n = 3. Adjust *p-values* are indicated. **C.** Immunoblot analysis of BCL-2, HEXIM1 and SKA2 in Z-138 cells treated with GSK-591, Ro or combination for 72h. **D.** Immunoblot analysis of the indicated proteins from Z-138 cells transduced with shRNAs targeting PRMT5 and MSI2, after 3 days of puromycin selection. **E.** Immunohistochemistry images showing that MSI2 depletion decreased c-MYC and BCL-2 protein abundance in tumors from mice treated with GSK-025 (100 mg/kg, BID 3x/week PO) for 3 weeks. Scale bars, 1000μm. **F.** Immunoblot showing the efficient BCL-2 knockout in Z-138 and OCI-LY19 Cas9 cells and the effect of GSK-591 and Ro (5μM) treatment for 72 h. **G.** Immunoblot analysis of Flag-BCL-2 expression in Z-138 cells overexpressing empty vector or Flag-BCL-2 72h post-transduction. **H.** Bar graph showing the fold change in IC50 values of GSK-591 (upper graph) and Venetoclax (bottom graph) in combination compared to single-agent. Shaded area showing the cell lines where the drug combination yielded > 5-fold shift in IC50 over single-agent, that represents strong synergy. Lymphoma cell lines were treated with PRMT5 inhibitor and venetoclax single agent or in combination using a fixed 1:1 ratio of each inhibitor over a 20-point titration, using a top dose of ≥14μM for both drugs. Cells were treated for 6 days and Celltiter-Glo proliferation assays were performed. **I.** Representative flow cytometry plots and quantification to show apoptosis of JVM-2 and Rec-1 cells treated with GSK-591 and/or venetoclax. Experiments were performed in triplicates and *p-values* were determined using two-tailed *t* test. **J.** Variations of body weight over time are shown from mice treated with vehicle, GSK-025 (50 mg/kg twice/day), venetoclax (100 mg/kg five times weekly) or the combination for 3 weeks.

Supplementary Tables

Supplementary Table T1. ClusterProfiler essential genes:

ID	Description	p.adjust	qvalue	Count	Gene.in.Pathway
GO:0016874	ligase activity	4.1E-08	3.3E-08	10	EARS2; GCLC; HARS2; LARS2; LIPT2; MARS2; PPCS; RTCB; VARS2; YARS2
GO:0004812	aminoacyl-tRNA ligase activity	4.1E-08	3.3E-08	6	EARS2; HARS2; LARS2; MARS2; VARS2; YARS2
GO:0016875	ligase activity, forming carbon-oxygen bonds	4.1E-08	3.3E-08	6	EARS2; HARS2; LARS2; MARS2; VARS2; YARS2
GO:0140101	catalytic activity, acting on a tRNA	2.0E-05	1.6E-05	7	EARS2; HARS2; LARS2; MARS2; RPP21; VARS2; YARS2
GO:0003735	structural constituent of ribosome	3.2E-05	2.6E-05	8	MRPL15; MRPL20; MRPL57; MRPS12; MRPS14; MRPS18A; MRPS34; MRPS6
GO:0140098	catalytic activity, acting on RNA	1.2E-03	9.9E-04	9	EARS2; HARS2; LARS2; MARS2; POLRMT; RPP21; RTCB; VARS2; YARS2
GO:0016853	isomerase activity	2.7E-02	2.1E-02	5	PGM3; RPE; RPIA; RPUSD4; TPI1
GO:0070181	small ribosomal subunit rRNA binding	2.7E-02	2.2E-02	2	MRPS18A; MRPS6
GO:0000049	tRNA binding	3.9E-02	3.2E-02	3	EARS2; HSD17B10; YARS2
GO:0048037	cofactor binding	3.9E-02	3.2E-02	8	ALAS1; COX15; ERCC2; GAPDH; GCLC; GLRX5; NUBP1; TKT
GO:0016779	nucleotidyltransferase activity	3.9E-02	3.2E-02	4	GMPPB; MTPAP; POLG2; POLRMT
GO:0016881	acid-amino acid ligase activity	3.9E-02	3.2E-02	2	GCLC; PPCS
GO:0019843	rRNA binding	3.9E-02	3.2E-02	3	MRPL20; MRPS18A; MRPS6
GO:0140097	catalytic activity, acting on DNA	3.9E-02	3.2E-02	5	ERCC2; N6AMT1; POLG2; RAD50; RUVBL1
GO:0051536	iron-sulfur cluster binding	3.9E-02	3.2E-02	3	ERCC2; GLRX5; NUBP1
GO:0051540	metal cluster binding	3.9E-02	3.2E-02	3	ERCC2; GLRX5; NUBP1
GO:0016780	phosphotransferase activity, for other substituted phosphate groups	4.2E-02	3.4E-02	2	AASDHPPT; CDIPT
GO:0008757	S-adenosylmethionine-dependent methyltransferase activity	4.7E-02	3.7E-02	4	DPH5; N6AMT1; PRMT5; WDR82
GO:0016769	transferase activity, transferring nitrogenous groups	4.7E-02	3.7E-02	2	GAPDH; GFPT1
GO:0008276	protein methyltransferase activity	5.0E-02	4.0E-02	3	N6AMT1; PRMT5; WDR82
GO:0004129	cytochrome-c oxidase activity	5.0E-02	4.0E-02	2	COX15; COX4I1
GO:0015002	heme-copper terminal oxidase activity	5.0E-02	4.0E-02	2	COX15; COX4I1
GO:0016676	oxidoreductase activity, acting on a heme group of donors, oxygen as acceptor	5.0E-02	4.0E-02	2	COX15; COX4I1
GO:0003678	DNA helicase activity	5.0E-02	4.0E-02	3	ERCC2; RAD50; RUVBL1

Supplementary Table T2. GSK-591 IC50 and TP53 mutation status

P53 STATUS	Lymphoma cells	IC50 (µM)	Allele frequency
WT	U2973	0.0086	1
WT	Z-138	0.024	1
X224_splice	SUDHL-6	0.053	0.49
Y234C	SUDHL-6	0.053	0.5
WT	OCI-LY19	0.058	1
G112_V122del	L-428	0.13	0.28
D281E	Maver-1	0.12	1
K132R	Ri-1	0.137	0.48
E294*	Ri-1	0.137	0.51
C176Y	U-2932	0.14	1
L334P	U-2932	0.14	0.09
WT	JVM-2	0.17	1
I254V	Ramos	0.25	0.99
I254N	Ramos	0.25	0.99
WT	SUP-M2	0.32	1
Deletion	HDLM2	0.45	Homozygous
R273C	Karpas-299	0.465	1
Y234N	SUDHL-8	0.52	0.47
R249G	SUDHL-8	0.52	0.52
R213Q	Raji	1.43	0.46
Y234H	Raji	1.43	0.57
G266E	Daudi	1.84	0.45
WT	TMD-8	2.9	1
WT	OCI-LY10	5.4	1
R282G	PDX 98848	Sensitive	1
P58Qfs*65	Jeko-1	7.86	0.97
R273C	SUDHL-4	9.24	1
H193R	BJAB	10.3	0.5
WT	OCI-LY3	11.2	1
R273H	SUDHL-1	11.7	0.66
WT	JVM-13	12.6	1
V147G	Mino	14.3	1
V157A	HBL-1	14.9	0.99
G244D	EB-1	16.5	1
G245D	Rec-1	17.4	0.51
Q317*	Rec-1	17.4	0.51
R248Q	CA-46	19.3	1
R248Q	DB	19.72	0.67
R248W	DB	19.72	0.34
R248Q	NUDHL-1	23.8	1
R248W	PDX 44685	Resistant	1

Supplementary Table T3. EnrichR Ro-targets Oncogenic Signatures

Term	Overlap	P.value	Adjusted.P.value	Old.P.value	Old.Adjusted.P.value	Odds.Ratio	Combined.Score
TBK1.DF DN	68/287	7.42E-16	1.35E-13	0	0	3.5782725	124.6554374
PIGF UP.V1 UP	52/191	4.75E-15	4.32E-13	0	0	4.28662956	141.3754874
VEGF A UP.V1 DN	45/193	1.03E-10	6.25E-09	0	0	3.46701586	79.72816825
GCNP SHH UP EARLY.V1 UP	34/174	1.66E-06	7.55E-05	0	0	2.75147477	36.61839395
CAMP UP.V1 DN	37/200	2.33E-06	8.47E-05	0	0	2.57330428	33.37693622
HOXA9 DN.V1 DN	36/195	3.33E-06	0.000101114	0	0	2.56570878	32.35746218
E2F1 UP.V1 UP	33/189	2.78E-05	0.000721913	0	0	2.3930535	25.10720594
GCNP SHH UP LATE.V1 UP	30/183	0.00020275	0.004612574	0	0	2.21439693	18.83020057
ERB2 UP.V1 DN	31/197	0.00033773	0.006829633	0	0	2.10881515	16.85632146
LTE2 UP.V1 DN	30/196	0.00066951	0.012185095	0	0	2.03952286	14.90679784
YAP1 UP	Nov-47	0.00121084	0.020033864	0	0	3.43248859	23.05411275
RB P130 DN.V1 DN	22/139	0.00214375	0.032513504	0	0	2.11724174	13.01087352
MEK UP.V1 DN	28/196	0.002825	0.039549941	0	0	1.87835605	11.02453828
MTOR UP.N4.V1 DN	26/193	0.00843583	0.10235473	0	0	1.75255677	8.368926922
EGFR UP.V1 DN	26/196	0.01023116	0.116379485	0	0	1.72134537	7.8877501

Supplementary Table T4. P53 knockin primers and sgRNA sequences

TP53 R248W mutation knock-in

gRNA Sequences

gRNA label	gRNA sequence 5' - 3'
gRNA_1	CCGGTTCATGCCGCCCATGC
gRNA_2	GCATGGGCGGCATGAACCGG
gRNA_3	CCTGCATGGGCGGCATGAAC

**Repair Template Sequence**
C*T*GACCTGGAGTCTTCCAGTGTGATGATGGTGAGGATGGGTCTCCAATTCATGCCGCCCATGCAGGAACTGTTACACATGTAGTTGT*A*G
* Phosphorothioated DNA bases

**Primer List**		
**Application**	**primer name**	**Sequence 5'-3'**
Amplicon sequencing	p53ex7F1	ACCATCCACTACAACTACATGTGTAACAGTTC
Amplicon sequencing	p53intR1	TAGTAGTATGGAAGAAATCGGTAAGAGGTGG
Sanger sequencing	p53ex6F2	AGCCGCCTGAGGTCTGGTTTGCAACTG
Sanger sequencing	p53intR2	ATGTGATGAGAGGTGGATGGGTAGTAGTATGG
Sanger sequencing	M13R2	CAGGAAACAGCTATGACC
