# Supplementary material for: TP53 mutations and RNA-binding protein MUSASHI-2 drive resistance to PRMT5-targeted therapy in B-cell lymphoma
Source: Nat Commun. 2022 Sep 27;13:5676. doi: 10.1038/s41467-022-33137-8 (PMC9515221; doi:10.1038/s41467-022-33137-8)
Supplement: Supplementary file 3 — Reporting Summary [file 41467_2022_33137_MOESM3_ESM.pdf]

nature portfolio

Corresponding author(s): Michael Kharas and Anas Younes

Last updated by author(s): Aug 25, 2022

Reporting Summary

Nature Portfolio wishes to improve the reproducibility of the work that we publish. This form provides structure for consistency and transparency in reporting. For further information on Nature Portfolio policies, see our Editorial Policies and the Editorial Policy Checklist.

Statistics

For all statistical analyses, confirm that the following items are present in the figure legend, table legend, main text, or Methods section.

n/a	Confirmed	
☐	☒	The exact sample size (*n*) for each experimental group/condition, given as a discrete number and unit of measurement
☐	☒	A statement on whether measurements were taken from distinct samples or whether the same sample was measured repeatedly
☐	☒	The statistical test(s) used AND whether they are one- or two-sided<br>*Only common tests should be described solely by name; describe more complex techniques in the Methods section.*
☒	☐	A description of all covariates tested
☐	☒	A description of any assumptions or corrections, such as tests of normality and adjustment for multiple comparisons
☐	☒	A full description of the statistical parameters including central tendency (e.g. means) or other basic estimates (e.g. regression coefficient) AND variation (e.g. standard deviation) or associated estimates of uncertainty (e.g. confidence intervals)
☐	☒	For null hypothesis testing, the test statistic (e.g. *F*, *t*, *r*) with confidence intervals, effect sizes, degrees of freedom and *P* value noted<br>*Give P values as exact values whenever suitable.*
☒	☐	For Bayesian analysis, information on the choice of priors and Markov chain Monte Carlo settings
☒	☐	For hierarchical and complex designs, identification of the appropriate level for tests and full reporting of outcomes
☐	☒	Estimates of effect sizes (e.g. Cohen's *d*, Pearson's *r*), indicating how they were calculated

Our web collection on statistics for biologists contains articles on many of the points above.

Software and code

Policy information about availability of computer code

Data collection	All software used for data collection are commercially available and stated within the Methods section (with detailed information of veersion used). Of note, for RNA-seq: Illumina HiSeq platform was used. For cell sorting, a BS FACS Aria II equipped with BD FACSDiva software (v8.0) was utilized (November 2008 edition).
Data analysis	EdgeR v3.0 (Empirical Analysis of digital expression data in R), ENRICHR 2013 (gene pathway enrichment analysis, developed by the Ma'ayan lab), GSEA v3.0 (Gene set enrichment analysis) and HOMER v4.10 for motif analysis. GraphPad Prism v8.0 was also used for analysis of various experiments and FlowJo v10.4 was used for sorting and flow cytometry analysis.

For manuscripts utilizing custom algorithms or software that are central to the research but not yet described in published literature, software must be made available to editors and reviewers. We strongly encourage code deposition in a community repository (e.g. GitHub). See the Nature Portfolio guidelines for submitting code & software for further information.

Data

Policy information about availability of data

All manuscripts must include a data availability statement. This statement should provide the following information, where applicable:

- Accession codes, unique identifiers, or web links for publicly available datasets
- A description of any restrictions on data availability
- For clinical datasets or third party data, please ensure that the statement adheres to our policy

All datasets generated in this study have been deposted in the gene expression Omnibus under the accession number GSE194363 and is available to the public.

Human research participants

Policy information about studies involving human research participants and Sex and Gender in Research.

Reporting on sex and gender	*Use the terms sex (biological attribute) and gender (shaped by social and cultural circumstances) carefully in order to avoid confusing both terms. Indicate if findings apply to only one sex or gender; describe whether sex and gender were considered in study design whether sex and/or gender was determined based on self-reporting or assigned and methods used. Provide in the source data disaggregated sex and gender data where this information has been collected, and consent has been obtained for sharing of individual-level data; provide overall numbers in this Reporting Summary. Please state if this information has not been collected. Report sex- and gender-based analyses where performed, justify reasons for lack of sex- and gender-based analysis.*
Population characteristics	*Describe the covariate-relevant population characteristics of the human research participants (e.g. age, genotypic information, past and current diagnosis and treatment categories). If you filled out the behavioural & social sciences study design questions and have nothing to add here, write "See above."*
Recruitment	*Describe how participants were recruited. Outline any potential self-selection bias or other biases that may be present and how these are likely to impact results.*
Ethics oversight	*Identify the organization(s) that approved the study protocol.*

Note that full information on the approval of the study protocol must also be provided in the manuscript.

Field-specific reporting

Please select the one below that is the best fit for your research. If you are not sure, read the appropriate sections before making your selection.

☒ Life sciences ☐ Behavioural & social sciences ☐ Ecological, evolutionary & environmental sciences

For a reference copy of the document with all sections, see nature.com/documents/nr-reporting-summary-flat.pdf

Life sciences study design

All studies must disclose on these points even when the disclosure is negative.

Sample size	For in vitro cell viability assays, Western blots, qPCR, RIP, and luciferase reporter assays we aimed for a minimum of 3 independent experiments peer group to allow for basic statistical inference. Genome-wide and validation CRISPR screens were performed in two biological replicas. For in vivo drug studies, at least five mice per group were included in order to achieve an alpha value of 0.8. HyperTRIBE and RNA-seq were performed in three biological replicates.
Data exclusions	No data were excluded from analysis.
Replication	All replication attempts were successful. For reproducibility of the CRISPR screens in Z138s and OCILY19 cells, and HyperTRIBE assays in Z138s, we used Pearson correlation analysis of normalized sgRNA counts and indicated in the manuscript the high replicate reproducibility in all experimental conditions based on the r correlation coefficient. the number of independent biological repeats performed for each experimeny are indicated through the manuscript main content, methods, and figure legends. Experiments were repeated at least twice by different scientists in the lab.
Randomization	Randomization was utilized for the in vivo drug experiments. Recipient mice were allocated into different groups randomly. For other experiments, all samples were treated in the same way to decrease the variability.
Blinding	No experiments were blinded as it wasn't necessary.

Reporting for specific materials, systems and methods

We require information from authors about some types of materials, experimental systems and methods used in many studies. Here, indicate whether each material, system or method listed is relevant to your study. If you are not sure if a list item applies to your research, read the appropriate section before selecting a response.

Materials & experimental systems

n/a	Involved in the study
☐	☒ Antibodies
☐	☒ Eukaryotic cell lines
☒	☐ Palaeontology and archaeology
☐	☒ Animals and other organisms
☒	☐ Clinical data
☒	☐ Dual use research of concern

Methods

n/a	Involved in the study
☒	☐ ChIP-seq
☐	☒ Flow cytometry
☒	☐ MRI-based neuroimaging

Antibodies

Antibodies used	PRMT5 (cat. ab31751, clone NA, dilution 1:1000), MSI2 (cat. ab76148, clone EPY1305Y, dilution 1:2000), H3R2me2 (cat. ab194684, dilution 1:1000), H4R3me2 (cat. ab5823, dilution 1:500), HEXIM1 (cat. ab25388, dilution 1:1000) and NOXA (cat. ab13654, clone 114C307, dilution 1:500) were purchased from Abcam. P53 (cat. 2527, clone 7F5, dilution 1:1000), c-MYC (cat. 9402, clone, D84C12, dilution 1:1000), BCL-2 (cat. 4223, clone 4223S, dilution 1:5000), BAX (cat. 2774, dilution 1:1000), MEP50 (cat. 2823, dilution 1:1000), SmD3me2 (cat. 13222, dilution 1:1000), PARP (cat. 9542, dilution 1:1000), Cleaved caspase-3 (cat. 9661, dilution 1:1000), P21 (cat. 2947, dilution 1:5000), PUMA (cat. 12450, clone, D30C10, dilution 1:1000), MDM2 (cat 86934, clone D1V2Z, dilution 1:1000) CYCLIN B1 (cat. 12231, clone D5C10, dilution 1:1000), CDK4 (cat. 12790, clone D9G3E, dilution 1:1000), CHK1 (cat. 2360, clone 2G1D5, dilution 1:1000), RAD51 (cat. 8875, clone D4B10, dilution 1:500), GFP (cat. 2956, dilution 1:1000) and α-TUBULIN (cat. 3873, clone YL1/2, dilution 1:10000) were purchased from Cell Signaling Technology. β-ACTIN (cat. 5316, dilution 1:10000) and anti-FLAG (cat. F3165, dilution 1:5000) were purchased from Sigma-Aldrich. SKA2 (cat. PA5-20818, dilution 1:500) was purchased from Invitrogen.
Validation	All antibodies have been validated for the indicated application by the manufacturers.

Eukaryotic cell lines

Policy information about cell lines and Sex and Gender in Research

Cell line source(s)	Human MCL-derived cell lines Z-138, Rec-1, Jeko-1, Mino-21, JVM-2, JVM-13, and BLCL-derived cell lines Raji, EB-1, Daudi, Ramos, and CA-46, were obtained from ATCC (Ameerican Type Culture Collection). HEK293T cells were also obtained from ATCC. Human DLBCL-derived cell lines SUDHL-4, SUDHL-6, SUDHL-8, SUDHL-10, OCI-LY19, DB, NUDHL-1, U-2973, OCI-LY3, U-2932, Ri-1, and OCI-LY10, Hodgkin's cell lines L-428, HDLM-2, and the MCL cell line, Maver-1, wer eobtained from the DSMZ-German Collection of microorganisms and Cell Cultures, Department of Human and Animal Cell Cultures (Braunschweig, Germany). Cell lines HBL-1, TMD-8, SUP-M2, SUDHL-1, Karpas-299, and BJAB are not commercially available, and were kindly provided by Dr. R. E. Davis from the MD Anderson Cancer Center in Houston TX. P-4936 cells (Pajic et al. 2000; Zellet et al. 2006) were provided by Dr. J. Zhang (Thompson Lab, Memorial Sloan Kettering Cancer Center).
Authentication	Cell lines were autheniticaed by STR analysis at the Integrated Genomic Operation Core Facility at Memorial Sloan KEttering Cancer Center, New York, NY.
Mycoplasma contamination	Cells were routinely tested and confirmed negative for mycoplasma in house using the mycoplasma detection kit available from Lonza Biosciences (cat.#LT07-218) according to manufacturer's instructions.
Commonly misidentified lines (See ICLAC register)	No commonly misidentified cell lines were used in this study.

Animals and other research organisms

Policy information about studies involving animals; ARRIVE guidelines recommended for reporting animal research, and Sex and Gender in Research

Laboratory animals	6-week old female NSG mice (NOD SCID Gamma) were used for the xenograft studies. Housing conditions are provided in the methods section.
Wild animals	No wild animals were used in this study.
Reporting on sex	We performed experiments with cell lines and patient samples that represent both sexes
Field-collected samples	No field-collected samples were used in this study.

Ethics oversight	All animal studies were conducted on animal protocols approved by the Institutional Animal Care and Use Committee (IACUC) at Memorial Sloan Kettering Cancer Center and GSK policies on the Care, Welfare, and Treatment of Laboratory Animals.

Note that full information on the approval of the study protocol must also be provided in the manuscript.

Flow Cytometry

Plots

Confirm that:

- [x] The axis labels state the marker and fluorochrome used (e.g. CD4-FITC).
- [x] The axis scales are clearly visible. Include numbers along axes only for bottom left plot of group (a 'group' is an analysis of identical markers).
- [x] All plots are contour plots with outliers or pseudocolor plots.
- [x] A numerical value for number of cells or percentage (with statistics) is provided.

Methodology

Sample preparation	Lymphoma cells were transduced with GFP-expressing lentivirus encoding MSI2 shRNA, or sgRNAs targeting P53 or BCL-2. Cells were centrifuged for 5 min at 1500 rpm, and washed twice with PBS. Viable cell numbers were determined using Trypan Blue staining. For CRISPR knock-out cell line generation, cells were single-cell sorted.
Instrument	Aria II Flow cell sorter from BD Biosciences (November 2008 edition) quipped with BD FACSDiva software (v8.0) was used for cell sorting.
Software	FlowJo Software v10.4 was used for FACS data analysis.
Cell population abundance	Purity of sorted cells was determined via the flow cytometer after sorting samples with purity higher than 95% were used.
Gating strategy	Gating Strategies are provided in the source data file. For sorting cells, Cells were gated as viable on the FSA by SSA gate, then for single cells by SSW and SSH and again by FSW and FSH. Then using positive and negative controls and GFP positive gate was created from the single cell gates and cells falling within the GFP positive gate were collected. GFP positive gate was drawn within SSA by FITC axes. For cell cycle analysis, gates were drawn on Propidium iodide (PI staining) as a histogram plot. Apoptosis was assesed by staining cells with Propidium Iodide (PI) and Annexin V and gated as such.

- [x] Tick this box to confirm that a figure exemplifying the gating strategy is provided in the Supplementary Information.
