# Supplementary material for: TP53 mutations and RNA-binding protein MUSASHI-2 drive resistance to PRMT5-targeted therapy in B-cell lymphoma
Source: Nat Commun. 2022 Sep 27;13:5676. doi: 10.1038/s41467-022-33137-8 (PMC9515221; doi:10.1038/s41467-022-33137-8)
Supplement: Supplementary file 4 — Description of Additional Supplementary Data Files [file 41467_2022_33137_MOESM4_ESM.pdf]

Description of Additional Supplementary Files

File Name: Supplementary Data 1. Essential genes for lymphoma survival

Description: Table containing genes, and their corresponding log fold changes and p-values found to be necessary for lymphoma cell line survival.

File Name: Supplementary Data 2. GSK-591 Genome-wide CRISPR screen

Description: Table of genes from the CRISPR screen in Figure 1, that, when deleted, confer sensitivity to GSK-591 treatment and their corresponding log fold changes, p-values, and FDRs.

File Name: Supplementary Data 3. Validation CRISPR OCI-LY19

Description: Repeat of genome-wide CRISPR screen done in Figure 1 and in second lymphoma cell line to validate hits

File Name: Supplementary Data 4. GSEA P53 pathway and ribonucleoprotein complex

Description: Table of shared genes generated from running Gene Set Enrichment Analysis of CRISPR screen hits against Hallmark Targets of p53 gene set and GOBP Ribonucleoprotein complex biogenesis gene set.

File Name: Supplementary Data 5. Heatmap DeSeq log2FC_adj

Description: Table of those genes represented in the heatmap found in Figure 4, the heatmap showing differential expression of the top 2600 significantly enriched (in red) and depleted (blue) transcripts in cells treated with 5uM of Ro, GSK-591 alone or in combination

File Name: Supplementary Data 6. MSigDB Enrichr DeSeq Combo vs Ro

Description: Enrichr results of genes upregulated or downregulated in reponse to combination GSK-591 and Ro treatment and Ro single treatment.

File Name: Supplementary Data 7. GSEA Combo vs Ro P53

Description: GSEA of DeSeq data of combination vs. Ro data. Table of shared genes generated from running Gene Set Enrichment Analysis of hits against Hallmark Targets of p53 gene set and hypoxia.

File Name: Supplementary Data 8. GSEA Downregulated genes

Description: GSEA of DeSeq data of combination vs. Ro data. Table of shared genes generated from running Gene Set Enrichment Analysis of hits against Hallmark Targets of Hallmark Targets of Myc V1, Hallmark E2F targets, and hypoxia.
File Name: Supplementary Data 9. Heatmap max.edit.freq
Ro.vs.Control_DMSO_Ro_GSK591_GSK591.Ro_shared

Description: Table of edit frequencies of MSI2 hyperTRIBE in response to single treatment of DMSO, Ro, and GSK-591 and dual treatment.

File Name: Supplementary Data 10. EnrichR Ro-targets GO molecular Functions

Description: Ro-specific MSI2 targets were run through Enrichr program which match different functions in molecular processes.

File Name: Supplementary Data 11. Overlap DeSeq, Ro-targets and CRISPR resistant genes

Description: Gene list of those genes found in the triple overlap Venn diagram, shared and not shared.
